# A novel selective fluorescent chemosensor for Fe ^3+^ ions based on phthalonitrile dimer: synthesis, analysis, and theoretical studies

**DOI:** 10.3906/kim-2004-68

**Published:** 2020-10-26

**Authors:** Shaya AL-RAQA, İpek ÖMEROĞLU, Doğan ERBAHAR, Mahmut DURMUŞ

**Affiliations:** 1 Department of Chemistry, Faculty of Science, Taibah University, Al-Madinah Al Munawarah Saudi Arabia; 2 Department of Chemistry, Faculty of Basic Sciences, Gebze Technical University, Kocaeli Turkey; 3 Department of Mechanical Engineering, Faculty of Engineering, Doğuş University, İstanbul Turkey

**Keywords:** Phthalonitrile, Suzuki cross-coupling, fluorescence, chemosensor, Fe ^3+^ ion

## Abstract

Phenyl-4,4-di(3,6-dibutoxyphthalonitrile) ( **3** ) was synthesized by the reaction of 1,4-phenylenebisboronic acid ( **1** ) and 4-bromo-3,6-dibutoxyphthalonitrile ( **2** ), using Suzuki cross-coupling reaction. The newly synthesized compound ( **3** ) was characterized by FT-IR, MALDI-MS, ESI-MS, ^1^ H-NMR, ^13^ C-NMR, and ^13^ C-DEPT-135-NMR. The fluorescence property of phenyl-4,4-di(3,6-dibutoxyphthalonitrile) ( **3** ) towards various metal ions was investigated by fluorescence spectroscopy, and it was observed thatthe compound ( **3** ) displayed a significantly ‘turn-off’ response to Fe ^3+^ , which was referred to 1:2 complex formation between ligand ( **3** ) and Fe ^3+^ . The compound was also studied via density functional theory calculations revealing the interaction mechanism of the molecule with Fe ^3+^ ions.

## 1. Introduction

Among the most abundant and versatile transition metal ions, iron is of vital importance to living systems because of its significant role in biochemical processes, such as oxidoreductase catalysis, oxygen transport, and electron transport [1–3]. In the case of high or low levels of Fe ^3+^ , critical side effects and diseases can be develop in the human body [1]. Deficiency of Fe ^3+^ may lead to low oxygen delivery to cells, resulting in diseases, such as anemia, liver disease, cancer, or diabetes [4]. On the other hand, excessive level of Fe ^3+^ can bring about some severe diseases, such as β-thalassemia, Friedreich’s ataxia, and Alzheimer’s disease [5]. Iron is important not only for biochemical processes, but also for the environment. Industrial waste that includes heavy metal ions (Cd, Cr, Cu, Fe, Hg, Pb, etc) is the biggest cause of environmental pollution. Determination and analysis of these heavy metal ions have gained importance for biology and environmental chemistry [6]. Atomic absorption spectrometry (AAS), flow-injection chemiluminescence, spectrophotometry, inductively coupled plasma-optical emission spectrometry/mass spectrometry (ICP-OES/MS), fluorimetry, and cathodic stripping voltammetry are used for the determination of the Fe ^3+^ and Fe ^2+^ ions, but these methods suffer from restrictions like using multifaceted instruments and interference with other metals [7–8]. In other aspects, fluorescence sensor is a promising method for detection because of its high selectivity, cost-efficiency, real time reaction, and its simple operational process [9]. Based on the abovementioned benefits, this study focuses on the synthesis of the new fluorescence chemosensor phthalonitrile derivative (3) for the determination of the Fe ^3+^ ions.

In this work, phenyl-4,4-di(3,6-dibutoxyphthalonitrile) (3) was synthesized and characterized by spectroscopic methods, including FT-IR, MALDI-MS, ESI-MS, ^1^ H-NMR, ^13^ C-NMR, and ^13^ C-DEPT-135-NMR. Chemosensor behavior of this phthalonitrile compound (3) was determined towards different metal ions (Ag ^+^ , Al ^3+^ , Ba ^2+^ , Ca ^2+^ , Cd ^2+^ , Co ^2+^ , Cr ^3+^ , Cs ^+^ , Cu ^2+^ , Fe ^2+^ , Fe ^3+^ , Hg ^2+^ , K ^+^ , Li ^+^ , Mg ^2+^ , Mn ^2+^ , Na ^+^ , Ni ^2+^ , Pb ^2+^ , Zn ^2+^ ) by using fluorescence spectroscopy in solution. All these various cations were tested, and it was demonstrated that a turn-off chemosensor response was achieved towards Fe ^3+^ selectively. When the newly synthesized phenyl-4,4-di(3,6-dibutoxyphthalonitrile) (3) was compared with the compounds showing fluorescence sensor properties against Fe ^3+^ and Fe ^2+^ ions in the literature [10,11], this phthalonitrile compound (3) showed fluorescence sensor only against Fe ^3+^ . Thus, Fe ^3+^ ion can be detected directly in the presence of the mixture of Fe ^2+^ and Fe ^3+^ ions. Also, optimized geometry and the reasonable Fe ^3+^ complex structure of the newly synthesized phthalonitrile compound (3) were defined by using quantum chemical computations.

## 2. Experimental analysis

### 2.1. Materials and equipment

All the reagents and solvents used for the synthesis of phthalonitrile compound (3) were of reagent-grade quality obtained from Sigma-Aldrich Corp. (St. Louis, MO, USA). AgNO ^3^ , AlCl ^3^ , BaCl ^2^ , CaCl ^2^ , CdCl ^2^ , CoCl ^2^ , CrCl ^3^ , CsCl, CuCl ^2^ , FeCl ^2^ , FeCl ^3^ , HgCl ^2^ , KCl, LiCl, MgCl ^2^ , MnCl ^2^ , NaCl, NiCl ^2^ , PbCl ^2^ , and ZnCl2 metal salts for the specification of sensor properties of the phthalonitrile compound (3) were obtained from Sigma-Aldrich. 4-Bromo-3,6-dibutoxyphthalonitrile (2) was synthesized according to the given procedure in the literature [12].

The FT-IR spectrum was recorded on a Perkin Elmer Spectrum 100 spectrophotometer. ^1^ H-NMR, ^13^ C-NMR, and ^13^ C-DEPT-135-NMR spectra were recorded on a (Bruker AVANCE 400 MHz) spectrometer (Bruker BioSpin Corp., Billerica, MA, USA). The mass spectra were obtained MALDI-MS (Bruker microflex LT MALDI-TOF MS) for phthalonitrile 3 and ESI-MS (Bruker Micro TOF-ESI/MS) for 3+Fe ^3+^ complex. Electronic absorption spectra were measured on a Shimadzu 2101 UV-Vis spectrophotometer. Fluorescence emission spectra were recorded on a Varian Eclipse spectrofluorometer (Agilent Technologies, Inc., Santa Clara, CA, USA) using 1 cm path length cuvette at room temperature, and slit width was adjusted as 5 nm.

## 2.2. Synthesis of phenyl-4,4-di(3,6-dibutoxyphthalonitrile) (3)

Suzuki cross-coupling reaction of 1,4-phenylenebisboronic acid (1) (0.020 g, 1.2 × 10 ^–4^ mol), 4-bromo-3,6-dibutoxyphthalonitrile (2) (0.119 g, 3.4 × 10 ^–4^ mol), sodium carbonate (0.143 g, 1.3 × 10 ^–3^ mol), and palladium-tetrakis(triphenylphosphine) (0.02 g, 1.7 × 10 ^–5^ mol) were stirred in the mixture of toluene, ethanol, and water (3:3:1 respectively, 7 mL) at refluxing temperature under nitrogen atmosphere for 72 h. After that, the reaction mixture was cooled to room temperature, and water (30 mL) was added. Then, the mixture was extracted with dichloromethane three times. The solvent was removed in vacuo to leave a dark-brown oil which was purified by silica gel filled column chromatography (eluting with THF/n-Hexane, 1:4) to give the pure title compound as a white powder.

Yield: 5.73 g (70%). FT-IR ν _max_ /cm-1: 3090–3055 (Aromatic-CH), 2957–2872 (Aliphatic-CH), 2227 (C≡N), 1575, 1496, 1459, 1285, 1196. ^1^ H-NMR (400 MHz; DMSO-d _6_ ): δ (ppm) 7.61 (s, 4H, Ar-H), 7.57-–7.51 (m, 2H, Ar-H), 4.17–4.14 (t, 8H, O-CH _2_ ), 1.74–1.68 (m, 8H, -CH _2_ ), 1.48–1.40 (m, 8H, -CH _2_ ), 0.96-0.92 (t, 12H, -CH _3_ ). ^13^ C-NMR (400 MHz; DMSO-d _6_ ): 14.07, 18.98, 30.84, 69.97, 103.22, 114.02, 121.06, 129.06, 129.42, 153.37. MALDI calc. as 618.78 (m/z) for C _38_ H _42_ N _4_ O _4_ ; found: 618.51 (m/z) as [M] ^+^ .

## 2.3. Chemosensor studies

Spectroscopic changes upon the addition of metal salts to the ligand solutions were enlisted using a fluorescence spectrophotometer. All fluorescence emission spectral studies were performed in DMSO solution (C = 1.0 µM) of the phthalonitrile compound (3) at room temperature, while the water solutions (C = 0.1 M) of the corresponding metal chlorides (nitrate derivative for Ag ion) were used as the source of metal ions. Spectra were routinely acquired at 25 °C in a 1 cm path length quartz cuvette with a volume of 2 mL by the addition of different metal (Ag ^+^ , Al ^3+^ , Ba ^2+^ , Ca ^2+^ , Cd ^2+^ , Co ^2+^ , Cr ^3+^ , Cs ^+^ , Cu ^2+^ , Fe ^2+^ , Fe ^3+^ , Hg ^2+^ , K ^+^ , Li ^+^ , Mg ^2+^ , Mn ^2+^ , Na ^+^ , Ni ^2+^ , Pb ^2+^ , Zn ^2+^ ) solutions [13]. The titration experiments of ligand in the presence of Fe ^3+^ were carried out with the freshly prepared stock solutions of ligand in DMSO and metal salts in water using a fluorescence spectrophotometer.

## 2.4. Computational method

Computational studies have been carried out in the framework of density functional theory [14,15] within the general gradient approximation (GGA) as implemented in the SIESTA code [16,17]. Perdew–Burke–Ernzerhof parametrization [18] was used for the exchange-correlation functional, and a double-ζ basis set augmented by polarization orbitals. The interaction between the core and valence electrons is handled by Troullier–Martins norm-conserving pseudopotentials [19] in their fully separable form [20].

In our calculations, to model isolated phthalonitrile molecule while using periodic boundary conditions, 10 Angstrom vacuum distance was maintained between the structures to eliminate any interactions. Charge density and potentials are determined on a real-space mesh that corresponds to the plane wave cutoff energy of 200 Ry. Optimized geometries are obtained in a conjugate-gradient algorithm without symmetry constraints until all force components on each atom are less than 0.01 eV/A.

## 3. Results and discussion

### 3.1. Synthesis and characterization

**Scheme 1:**
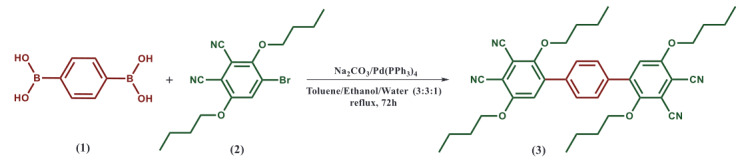
Synthetic pathway of phenyl-4,4-di(3,6-dibutoxyphthalonitrile) (3).

In this work, the phthalonitrile compound 3) was synthesized by the reaction of 1,4-phenylenebisboronic acid (1) with 4-bromo-3,6-dibutoxyphthalonitrile (2) by way of Suzuki cross-coupling reaction (Scheme 1). The newly synthesized phthalonitrile compound (3) was characterized by spectroscopic methods, including FT-IR, MALDI-MS, ESI-MS, ^1^ H-NMR, ^13^ C-NMR, and ^13^ C-DEPT-135-NMR.

In the FT-IR spectrum of the phthalonitrile compound (3), the vibration peaks were obtained between 3090 and 3055 cm ^−1^ belonging to aromatic-CH stretching, and between 2957 and 2872 cm ^−1^ belonging to aliphatic-CH stretching. As a result of Suzuki cross-coupling reaction, the broad peak of 1,4-phenylenebisboronic acid (1) for the -OH band disappeared, and the most characteristic peak for -C≡N stretching was observed as a sharp peak at 2227 cm ^−1^ for the phthalonitrile compound (3).

**Figure 1:**
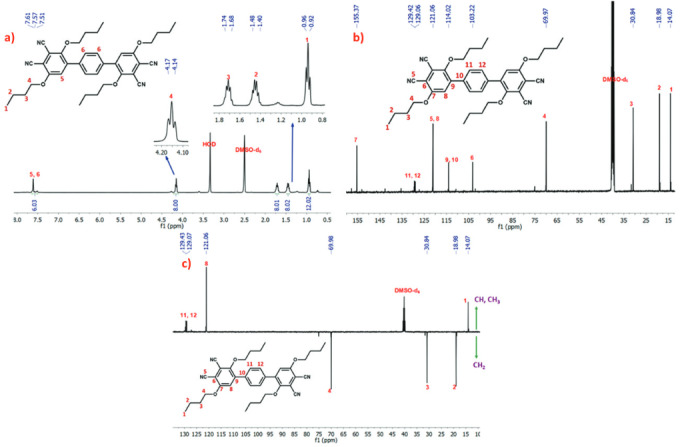
a) ^1^ H-NMR, b) ^13^ C-NMR, and c) ^13^ C-DEPT-135-NMR spectra of phenyl-4,4-di(3,6-dibutoxyphthalonitrile) (3) in DMSO-d _6_ .

The ^1^ H-NMR spectrum of the phthalonitrile compound (3) was measured in DMSO-d _6_ . This spectrum exhibited characteristic signals for the aromatic protons between 7.61 and 7.51 ppm. Besides, the aliphatic protons were displayed as a triplet peak at 4.15 ppm for -O-CH _2_ protons, as multiplet peaks between 1.71 and 1.44 ppm for -CH _2_ protons, and as a triplet peak at 0.94 for -CH _3_ protons (Figure 1a).

The ^13^ C-NMR spectrum of phthalonitrile compound (complied with the expected structure. ^13^ C-NMR spectrum is shown in Figure1b, which shows signals of aromatic carbons between 103.22 and 155.37 ppm, and aliphatic carbons between 14.07 and 69.97 ppm. The carbon atom of the -C≡N group on the phthalonitrile was observed at 121.06 ppm.

Figure 1c shows ^13^ C-DEPT-135 spectrum of phthalonitrile compound (3) in DMSO-d _6_ , and only the proton bearing carbons are defined at this spectrum. In DEPT 135 spectrum of this compound, 13C signals arising due to methyl (-CH _3_ ) and methine (-CH) appear positive, methylene as negative (-CH _2_ ) whereas no quaternary carbons show up.

**Figure 2:**
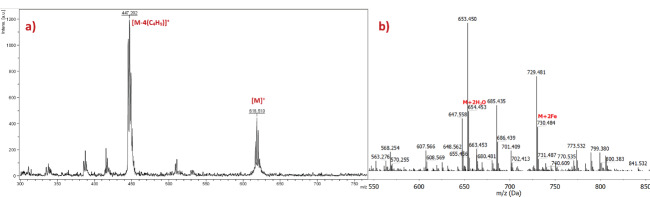
Mass spectra of phenyl-4,4-di(3,6-dibutoxyphthalonitrile) (3) a) before and b) after the addition of Fe ^3+^ solution.

The molecular ion peak of the phthalonitrile 3 was observed at 618.510 as [M] ^+^ by MALDI-TOF/TOF (Figure 2a). Two equivalents of Fe ^3+^ solution was added to phthalonitrile compound (3), which dissolved in acetone, for detecting metal-ligand complexes by using ESI-MS. The molecular ion peak of phthalonitrile compound (3) was observed 654.453 [M+2H _2_ O] ^+^ and 730.484 [M+2Fe] ^+^ after the addition of Fe ^3+^ solution (Figure 2b).

## 3.2. Chemosensor properties of phthalonitrile 3 to metal ions

**Figure 3:**
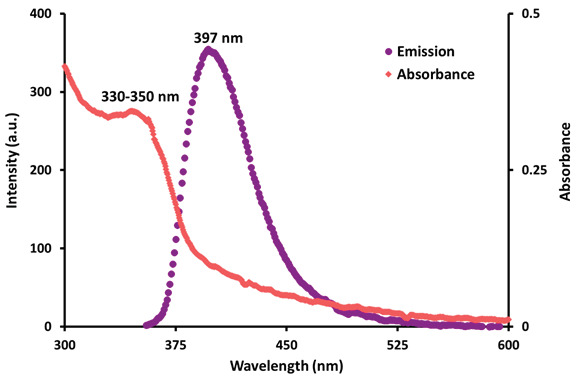
Absorption (C = 1.0 μM) and fluorescence emission (C = 2 μM) spectra of phthalonitrile compound (3) in DMSO (excitation wavelength = 340 nm).

The absorption and fluorescence properties of phthalonitrile compound (3) were studied in DMSO by UV-Vis absorption and fluorescence spectrophotometers, respectively. Broad absorption bands were observed between the ranges of 330–350 nm in the UV-Vis spectrum of this phthalonitrile compound (Figure 3). The fluorescence intensity maximum of phthalonitrile compound (3) was observed at 397 nm when excited at 340 nm in DMSO solution (Figure 3).

**Figure 4:**
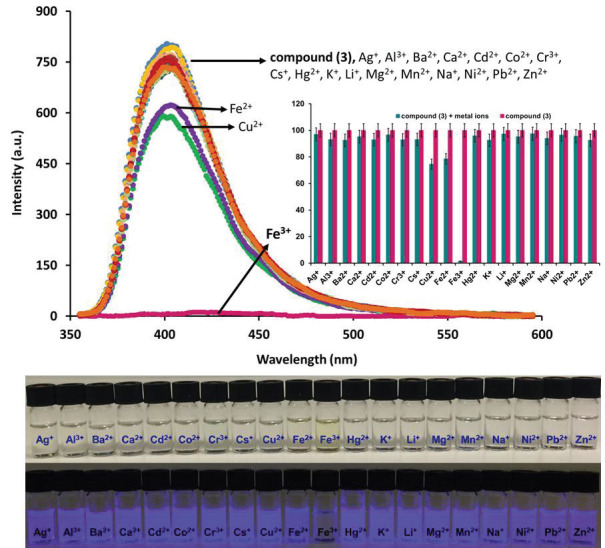
Fluorescence emission spectra of phthalonitrile compound (3) (C = 1.0 μM) after the addition of 500 μM different metal ions (excitation wavelength = 340 nm).

The chemosensor behavior of the phthalonitrile compound (3) was tested on a various of metal ions (Ag ^+^ , Al ^3+^ , Ba ^2+^ , Ca ^2+^ , Cd ^2+^ , Co ^2+^ , Cr ^3+^ , Cs ^+^ , Cu ^2+^ , Fe ^2+^ , Fe ^3+^ , Hg ^2+^ , K ^+^ , Li ^+^ , Mg ^2+^ , Mn ^2+^ , Na ^+^ , Ni ^2+^ , Pb ^2+^ , Zn ^2+^ ) using fluorescence spectrophotometer. For fluorescence emission spectral studies, the stock solution of phthalonitrile compound (3) was prepared in DMSO, and chloride salts of metal solutions (nitrate derivative for Ag ion) were prepared in water. 500 μM of different metal ions (Ag ^+^ , Al ^3+^ , Ba ^2+^ , Ca ^2+^ , Cd ^2+^ , Co ^2+^ , Cr ^3+^ , Cs ^+^ , Cu ^2+^ , Fe ^2+^ , Fe ^3+^ , Hg ^2+^ , K ^+^ , Li ^+^ , Mg ^2+^ , Mn ^2+^ , Na ^+^ , Ni ^2+^ , Pb ^2+^ , Zn ^2+^ ) were added to the solution of phthalonitrile compound (3) in DMSO. The fluorescence spectra of the phthalonitrile compound (3) exhibited little change after the addition of the 500 μM of metal solutions, except for Fe ^3+^ solution. Fluorescence intensity of the phthalonitrile compound (3) upon the addition of Fe ^3+^ showed a great quenching, but no or minimal change was displayed with other metal ions (Figure 4). Also, we observed a naked-eye detectable color change of this compound (3) to yellow after the addition of Fe ^3+^ . This phthalonitrile (3) showed ‘turn-off’ chemosensor properties against the Fe ^3+^ ions in the solution. The observed selectivity of the probe towards Fe ^3+^ over the Fe ^2+^ may be explained by taking into account the cavity size and radius of Fe ^3+^ ion [21].

**Figure 5:**
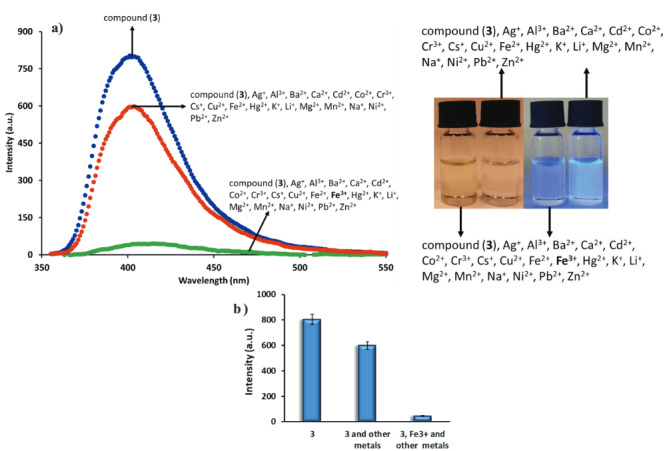
a) Fluorescence response of the phthalonitrile compound (3) (C=1.0 μM) to various cations in aqueous solution, b) histogram of fluorescence response for phthalonitrile compound (3) (excitation wavelength = 340 nm).

The change of the fluorescent intensity of phthalonitrile compound (3) is shown in Figure 5. Firstly, the blue line indicates the fluorescent intensity of the phthalonitrile compound (3) (C = 1.0 µM). The red line represents the fluorescence alters that take upon the addition of all the metals (Ag ^+^ , Al ^3+^ , Ba ^2+^ , Ca ^2+^ , Cd ^2+^ , Co ^2+^ , Cr ^3+^ , Cs ^+^ , Cu ^2+^ , Fe ^2+^ , Hg ^2+^ , K ^+^ , Li ^+^ , Mg ^2+^ , Mn ^2+^ , Na ^+^ , Ni ^2+^ , Pb ^2+^ , Zn ^2+^ ), except for Fe ^3+^ , the solution containing phthalonitrile compound (3). Finally, the green line shows the fluorescence changes that take upon the addition of all the metals(Ag ^+^ , Al ^3+^ , Ba ^2+^ , Ca ^2+^ , Cd ^2+^ , Co ^2+^ , Cr ^3+^ , Cs ^+^ , Cu ^2+^ , Fe ^2+^ , Hg ^2+^ , K ^+^ , Li ^+^ , Mg ^2+^ , Mn ^2+^ , Na ^+^ , Ni ^2+^ , Pb ^2+^ , Zn ^2+^ ), including Fe ^3+^ , the solution containing phthalonitrile compound (3).These results indicated that the selectivity of Fe ^3+^ ion was obtained in the presence of all the metals (Ag ^+^ , Al ^3+^ , Ba ^2+^ , Ca ^2+^ , Cd ^2+^ , Co ^2+^ , Cr ^3+^ , Cs ^+^ , Cu ^2+^ , Fe ^2+^ , Fe ^3+^ , Hg ^2+^ , K ^+^ , Li ^+^ , Mg ^2+^ , Mn ^2+^ , Na ^+^ , Ni ^2+^ , Pb ^2+^ , Zn ^2+^ ) and other metals (Ag ^+^ , Al ^3+^ , Ba ^2+^ , Ca ^2+^ , Cd ^2+^ , Co ^2+^ , Cr ^3+^ , Cs ^+^ , Cu ^2+^ , Fe ^2+^ , Hg ^2+^ , K ^+^ , Li ^+^ , Mg ^2+^ , Mn ^2+^ , Na ^+^ , Ni ^2+^ , Pb ^2+^ , Zn ^2+^ ), except for Fe ^3+^ ,which is significant for the detection of Fe ^3+^ ion.

**Figure 6:**
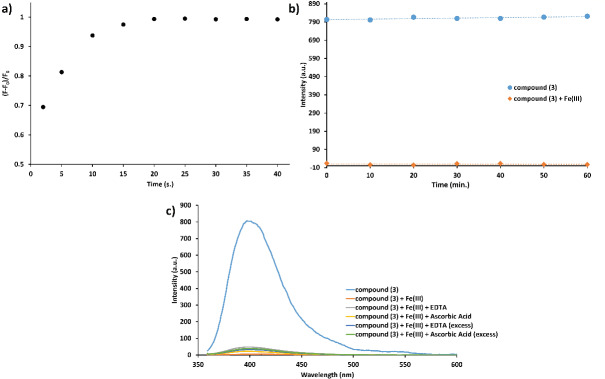
a) Effect of time on fluorescence signal, b) photostability, and c) reversibility test of the phthalonitrile compound (3) (C=1.0 μM) after the addition of 500 μM of Fe ^3+^ .

The effect of time on fluorescence signal change of phthalonitrile compound (3) for the detection of Fe ^3+^ was evaluated between 2–40 s (Figure 6a). The relative fluorescence intensity of phthalonitrile compound (3)-Fe ^3+^ complex was not found stable until the 20th s, and then it was nearly unchanged after this time. Therefore, this time is sufficient as the detection time of Fe ^3+^ for consistent and accurate results. The photostability of phthalonitrile compound (3) and its Fe ^3+^ complex was evaluated between 0 and 60 min at daylight (Figure 6b). Fluorescence signals of the phthalonitrile compound (3) and its Fe ^3+^ complex did not change and remained stable until the 60th min. The reversibility of the detection process of Fe ^3+^ with the phthalonitrile compound (3) was evaluated with EDTA and ascorbic acid after the complexation process (Figure 6c) [22]. The quenched fluorescence signal of the phthalonitrile compound (3) after the addition of Fe ^3+^ was not restored by the addition of EDTA and ascorbic acid, which showed that the Fe ^3+^ recognition is an irreversible process [23].

**Figure 7:**
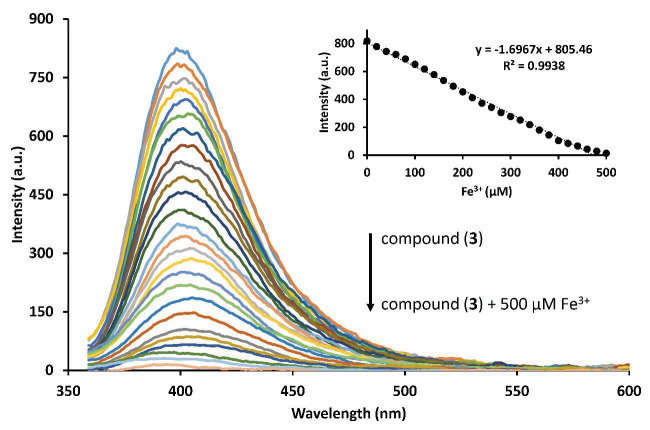
Fluorescence titration of the phthalonitrile compound (3) (C = 1.0 μM) after addition of a gradually increased amount of Fe ^3+^ . Inset: linear response change of the phthalonitrile compound (3) (C = 1.0 μM) after the addition of a gradually increased amount of Fe ^3+^ .

Fluorescence titration of the phthalonitrile compound (3) with Fe ^3+^ was performed to understand the binding mode of phthalonitrile compound (3) with Fe ^3+^ (Figure 7). For that purpose, a gradually increased amount of Fe ^3+^ up to 500 μM was added to DMSO solution of phthalonitrile compound (3). As can be seen in Figure 7, fluorescence signal of the phthalonitrile compound (3) which was observed at 397 nm, was proportionally decreased by the increased concentration of Fe ^3+^ , and finally it was completely quenched by the addition of 500 μM Fe ^3+^ . Linear response change of the phthalonitrile compound’s (3) fluorescence intensity by the addition of Fe ^3+^ at 397 nm is shown in Figure 7 inset, which was used for the calibration curve for Fe ^3+^ analysis. The linear regression equation was found as follows:

F= –1.6967[Fe ^3+^ ] + 805.46 for 2–500 μM ofFe ^3+^ (R ^2^ = 0.9938).

The limit of detection (LOD) and the limit of quantification (LOQ) were calculated according to 3σ/k and 9σ/k, respectively. These values were found as 3.28 μM and 9.85 μM, respectively, which pointed out high sensitivity [24,25]. Precision is an important parameter for fluorescence sensor applications. Therefore, the precision of the phthalonitrile compound (3) was determined using ten measurements for 500 μM Fe ^3+^ under optimum conditions, and the relative standard deviation (RSD%) was calculated as 2.81% for Fe ^3+^ .

**Figure 8:**
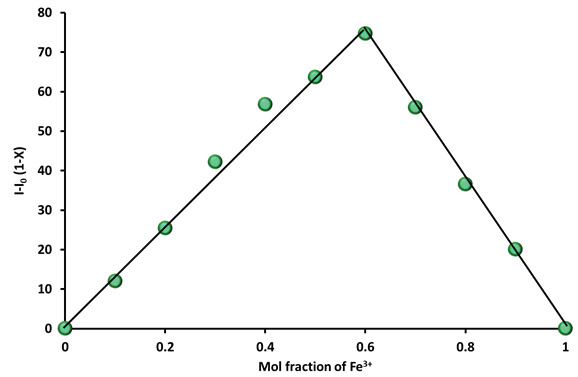
Job’s plot of 3-Fe ^3+^ complexes in DMSO solutions. The total concentration of 3 and Fe ^3+^ was 2 μM (excitationwavelength = 340 nm).

**Figure 9:**
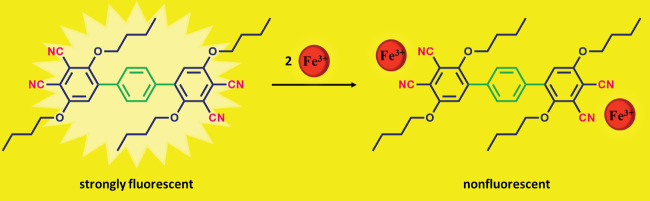
Schematic illustration for fluorescence turn-off sensing of Fe ^3+^ with the phthalonitrile compound (3).

The formation of the complex between phthalonitrile compound (3) (C = 2 µM) and Fe ^3+^ ion was experimentally indicated by performing a Job’s plot analysis. The titration of the phthalonitrile compound (3) with Fe ^3+^ cation showed a decrease in the fluorescence intensities by the increasing concentrations of Fe ^3+^ cation (C = 2 µM). In good agreement with theoretical calculations, the Job’s plot shows a maximum at 0.6 indication of a 1:2 stoichiometry between phthalonitrile 3 and Fe ^3+^ ion in DMSO (Figure 8). As it is expected, considering the Job’s plots and association constants in accordance with 1:2 stoichiometry, the possible binding mode between phthalonitrile compound (3) and Fe ^3+^ is proposed in Figure 9. The strong fluorescence intensity of this phthalonitrile 3 was turn-off after the addition of Fe ^3+^ ions.

After the investigation of binding properties of phthalonitrile compound (3) by the addition of Fe ^3+^ , the practical determination of Fe ^3+^ in industrial wastewater was carried out using spike/recovery test, and the results were calculated via calibration curve. As shown in Table 1, phthalonitrile compound (3) was found able to determine different concentrations of spiked Fe ^3+^ with good recovery, indicating that phthalonitrile compound (3) can potentially be employed for detecting Fe ^3+^ in real samples. In addition, analytical parameters of phthalonitrile compound (3) for Fe ^3+^ determination were compared with some other Fe ^3+^ - selective fluorescent sensor studies [26–30]. As seen in Table 2, the phthalonitrile compound (3) showed lower LOD and larger linear range than the given fluorescent sensors studied in the literature. This comparison demonstrated that the presented phthalonitrile compound (3) is a selective, sensitive, and an accessible alternative fluorescent sensor for the detection of Fe ^3+^ ions.

**Table 1:** Determination of Fe ^3+^ concentrations in the industrial wastewater sample.

Sample	Spikedconcentration (μM)	Found (μM)	Recovery (%)
Industrial wastewater	0	52.79 ± 0.15	-
	100	148.64 ± 1.15	95.85
	200	254.56 ± 2.02	100.88

**Table 2:** Comparison of the analytical data of the studied fluorescence chemosensor with previously reported chemosensors for iron sensing.

Material	Sample	Linear range (µM)	LOD (µM )	Ref.
4-aminobenzo-15-crown-5	Cancer cell imaging	–50.00-150.00	50.00	1
Gold Nanoclusters	Water samples and iron tablets	–5.00-1280.00	3.50	2
Rhodamine B derivative	Water samples	5.26	10–150	3
Julolidine derivatives	-	6.80	10–400	4
Benzothiazole derivative	Cell imaging	8.43 and 5.86	10–400 and 10–900	5
Phthalonitrile derivative	Water samples	2.00–500.00	3.28	In this work

## 3.3. Computational studies

**Figure 10:**
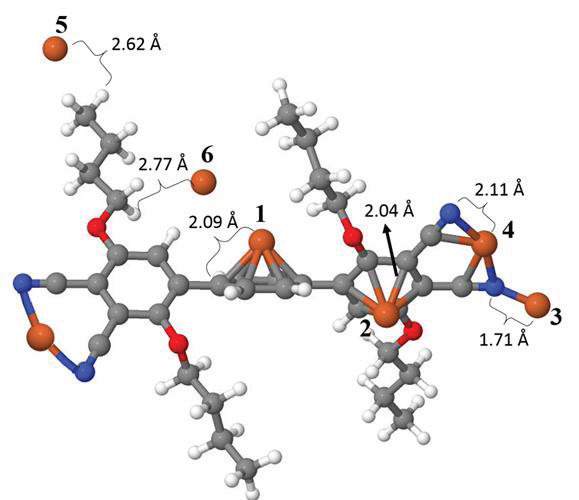

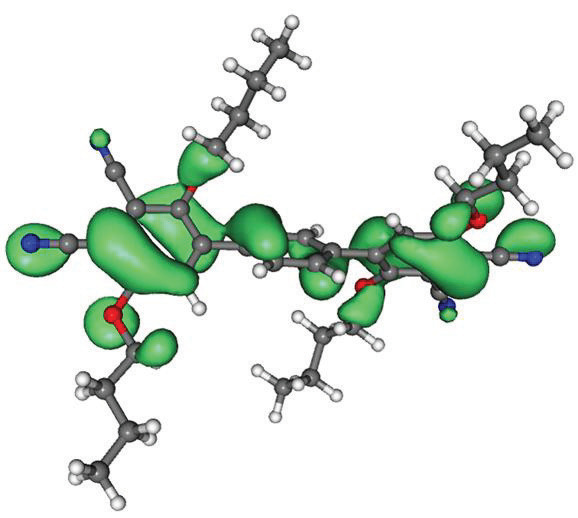
a) Ball and stick model of the phthalonitrile molecule. The possible interaction sites of Fe ^3+^ ion on the phthalonitrile molecule is marked by different numbers. The distances to the nearest atom on the molecule after the geometry optimization is also shown. b) A typical isosurface of HOMO orbital wavefunction (shown by a green balloon) of phthalonitrile molecule indicate the most active sites during a possible chemical interaction.

The interaction mechanism of Fe ^3+^ atom with phthalonitrile compound (3) was investigated via density functional theory. After optimizing the structure of the molecule, various possible interaction sites for Fe ^3+^ ion are scanned by placing a single Fe ^3+^ ion and letting the geometry optimize without any constraints. Figure 10a shows various possible interaction sites after the geometry optimization process. The adsorption energy for various sites are calculated using the formula E _ads_ = E(molecule + ion) ‒E(ion) ‒ E(molecule), where E(ion) and E(molecule) are the total energies of isolated ion and molecule, respectively, while E(molecule+ion) represents the total energy of the optimized molecule + ion system.

Table 3 shows the interaction energy values for different sites on the molecule. While energetically the most stable interaction site is found to be between the two cyanide groups, this site corresponds to a small fraction of the total surface area of the molecule. It is also worth to note that the interaction energy of site 4 is exactly twice as the energy of site 3, where Fe ^3+^ ion interacts with a single cyanide group, which indicates that the highest interaction energy of site 4 results from the duplication of a CN-Fe ^3+^ interaction, which is not as high as the hexagon-Fe ^3+^ interaction by itself. Moreover, the hexagons of the molecule constitute a significant fraction of the total surface area of the molecule, so it can be deduced that the most active sites of phthalonitrile molecule (3) are the ones around carbon rings. It is also clear that the Fe ^3+^ ion refuses to bind in sites 5 and 6, which are totally saturated carbon chains. Looking at the geometry, one can deduce that the interaction has a more physisorption character rather than a chemical activation.

We also investigated the electronic structure of the pure phthalonitrile molecule in the context of the frontier molecular orbital theory. A typical isosurface of HOMO state wavefunction is shown in Figure 10b, which is seen to be distributed over the active sites–especially carbon rings. This verifies our energetics analysis above.

**Table 3:** Interaction energies of Fe ^3+^ ion on phthalonitrile molecule for different interaction sites defined in Figure 10a.

Interaction site	Interaction energy (eV)
1	–2.47
2	–2.20
3	–1.62
4	–3.18
5	–0.41
6	–0.65

## 4. Conclusion

In summary, the novel phenyl-4,4-di(3,6-dibutoxyphthalonitrile) (3) was designed and synthesized as a new fluorescence chemosensor for Fe ^3+^ . This phthalonitrile compound (3) was characterized by different spectroscopic methods, such as FT-IR, MALDI-MS, ESI-MS, 1H NMR, 13C NMR, and ^13^ C-DEPT-135 NMR. In addition to these, the impacts of metal ions on the fluorescence attitude of the studied compound (3) were investigated to see whether this compound can be used as a chemosensors for metal ions. An important decrease in the fluorescence emission by the addition of the Fe ^3+^ cation was observed. Also, since change of color is observed in the solution when Fe ^3+^ was added, the perfect fluorescent response to Fe ^3+^ in DMSO can be detected even by the naked eye. Thanks to these results that were obtained, it can be asserted that this compound (3) designed as a metal-ion sensor has the potential to be used for a variety of chemical and biological applications in the future.
